# Identifying Pleiotropic SNPs Associated With Femoral Neck and Heel Bone Mineral Density

**DOI:** 10.3389/fgene.2020.00772

**Published:** 2020-07-22

**Authors:** Pei He, Xiang-He Meng, Xiao Zhang, Xu Lin, Qiang Zhang, Ri-Li Jiang, Martin R. Schiller, Fei-Yan Deng, Hong-Wen Deng

**Affiliations:** ^1^Center for Genetic Epidemiology and Genomics, School of Public Health, Medical College of Soochow University, Suzhou, China; ^2^Center for Bioinformatics and Genomics, School of Public Health and Tropical Medicine, Tulane University, New Orleans, LA, United States; ^3^Center of Reproductive Health, System Biology and Data Information, School of Basic Medical Science, Central South University, Changsha, China; ^4^Laboratory of Molecular and Statistical Genetics, College of Life Sciences, Hunan Normal University, Changsha, China; ^5^Department of Endocrinology and Metabolism, The Third Affiliated Hospital of Southern Medical University, Guangzhou, China; ^6^College of Public Health, Zhengzhou University, Zhengzhou, China; ^7^Nevada Institute of Personalized Medicine, School of Life Sciences, University of Nevada, Las Vegas, Las Vegas, NV, United States

**Keywords:** osteoporosis, cFDR, colocalization analysis, Mendelian randomization, pleiotropic, causal

## Abstract

**Background:**

Genome-wide association studies (GWASs) routinely identify loci associated with risk factors for osteoporosis. However, GWASs with relatively small sample sizes still lack sufficient power to ascertain the majority of genetic variants with small to modest effect size, which may together truly influence the phenotype. The loci identified only account for a small percentage of the heritability of osteoporosis. This study aims to identify novel genetic loci associated with DXA-derived femoral neck (FNK) bone mineral density (BMD) and quantitative ultrasound of the heel calcaneus estimated BMD (eBMD), and to detect shared/causal variants for the two traits, to assess whether the SNPs or putative causal SNPs associated with eBMD were also associated with FNK-BMD.

**Methods:**

Novel loci associated with eBMD and FNK-BMD were identified by the genetic pleiotropic conditional false discovery rate (cFDR) method. Shared putative causal variants between eBMD and FNK-BMD and putative causal SNPs for each trait were identified by the colocalization method. Mendelian randomization analysis addresses the causal relationship between eBMD/FNK-BMD and fracture.

**Results:**

We identified 9,500 (cFDR < 9.8E-6), 137 (cFDR < 8.9E-4) and 124 SNPs associated with eBMD, FNK-BMD, and both eBMD and FNK-BMD, respectively, with 37 genomic regions where there was a SNP that influences both eBMD and FNK-BMD. Most genomic regions only contained putative causal SNPs associated with eBMD and 3 regions contained two distinct putative causal SNPs influenced both traits, respectively. We demonstrated a causal effect of FNK-BMD/eBMD on fracture.

**Conclusion:**

Most of SNPs or putative causal SNPs associated with FNK-BMD were also associated with eBMD. However, most of SNPs or putative causal SNPs associated with eBMD were not associated with FNK-BMD. The novel variants we identified may help to account for the additional proportion of variance of each trait and advance our understanding of the genetic mechanisms underlying osteoporotic fracture.

## Introduction

Osteoporosis is a complex disease that prevalently occurs in postmenopausal women and is diagnosed primarily by measuring bone mineral density (BMD) (Tella and Gallagher, 2014). Low femoral neck (FNK) BMD is a major risk factor of osteoporosis and remains the best predictor of primary osteoporotic fractures (Meng et al., 2018). It is established that there are differences of genetic determination among various skeletal sites (Yang et al., 2006). The heritability of BMD is estimated between 46 and 84% depending on the skeleton site studied (Mullin et al., 2018).

Although BMD can be measured in many ways, the “gold standard” diagnostic technique is dual energy absorptiometry (DXA) of the spine and hip (Tella and Gallagher, 2014). However, DXA is relatively expensive, and consequently, the genome-wide association studies (GWASs) of DXA-derived BMD include a relatively small size of samples, compromising the ability to detect genetic loci. Quantitative ultrasound (QUS) of the heel calcaneus estimated BMD (referred to as estimated BMD “eBMD” in this study) is quick, safe, relatively inexpensive and is cost-effective for GWASs with very large samples of individuals (Kemp et al., 2017). The correlations of eBMD with spine (*r* = 0.50) and hip BMD (*r* = 0.54) were modest but stronger in postmenopausal women (*r* = 0.60 for spine and *r* = 0.62 for hip, respectively) (Saadi et al., 2004). Furthermore, eBMD has been used in combination with other clinical risk factors to predict the risk of fracture (Khaw et al., 2004).

Genome-wide association studies is a powerful tool to identify susceptibility genetic variants of complex diseases. One previous GWAS identified 56 BMD-associated loci, accounting for approximately 5.8% of BMD variance (Estrada et al., 2012). The conditionally independent genome-wide significant lead single nucleotide polymorphisms (SNPs) account for 20% of the total variance of eBMD (Morris et al., 2019). However, despite the relative large sample sizes, GWASs with these sample sizes may still lack sufficient power to identify the majority of genetic variants with small to modest effect sizes, which may truly influence the phenotype (Pei et al., 2014).

Pleiotropy is a phenomenon in which a genetic variant affects multiple phenotypes. The presence of pleiotropy indicates that related traits may also share some genetic determinants (Greenbaum et al., 2017). Identifying potential shared causal variants among traits can help determine the overlapping etiologies of multifactorial disorders. Therefore, we considered variants related to both BMD and normal bone physiology. There have been limited efforts to ascertain the mechanisms that alter bone physiology or the causal genes underlying the GWAS loci (Sabik and Farber, 2017). GWASs allow us to obtain the estimates of effect size for all those genetic variants, so it is possible to estimate shared genetic determinants by checking the correlations between the effect sizes across traits/bone parameters, which does not need to measure multiple traits in the same individuals (Bulik-Sullivan et al., 2015). We used GWAS-derived summary statistics to identify the shared genetic variants for two related phenotypes with the genetic pleiotropy-informed conditional false discovery rate (cFDR) method (Andreassen et al., 2013). This method incorporates the GWAS-derived summary statistics for two related phenotypes to test the association of genetic variants with one phenotype conditional on the different strengths of the association of these variants with the second phenotype (Greenbaum et al., 2017). cFDR improves the statistical power and identifies additional variants associated with each trait and pleiotropic variants from the studies of traits or diseases (Lv et al., 2017). Bayesian colocalization models identify genomic regions containing the same association signal by GWAS and colocalizing expression quantitative trait loci (eQTL) signals or GWAS signals of two traits (Giambartolomei et al., 2014; Pickrell et al., 2016). This model is the basis to integrally consider the effect sizes of all genetic variants in the pre-defined regions (e.g., approximately independent linkage disequilibrium (LD) blocks) (Berisa and Pickrell, 2016) and calculate the posterior probabilities of each region containing one causal variant associated with both traits (Giambartolomei et al., 2014; Pickrell et al., 2016).

In this study, we aim to compare those genetic loci associated with DXA-derived FNK-BMD or eBMD and putative causal SNPs associated with each trait. Novel variants with pleiotropic or causal effects on eBMD and FNK-BMD were identified by cFDR and colocalization analysis of GWAS summary statistics. Moreover, we investigated the relationship between fracture and FNK-BMD/eBMD by Mendelian randomization (MR) analysis of summary statistics in order to assess if FNK-BMD/eBMD causally underlie fracture risk.

## Materials and Methods

### GWAS Datasets

The summary statistics analyzed were derived from three publicly available online GWAS datasets: FNK-BMD dataset, eBMD dataset and fracture dataset. The FNK-BMD GWAS dataset contained the association results for approximately 10 million SNPs and is derived from a GWAS meta-analysis of several studies with 32,965 European subjects published by the Genetic Factors for Osteoporosis (GEFOS) Consortium (Zheng et al., 2015). A GWAS meta-analysis was conducted in 426,824 subjects of European ancestry to study the associations of over 14 million SNPs with eBMD (Morris et al., 2019). The fracture dataset was generated from a GWAS meta-analysis of 264,973 European subjects, containing summary statistics of more than 13 million SNPs (Morris et al., 2019).

### Data Preparation

We pruned SNPs in the FNK-BMD and eBMD datasets before cFDR analysis using the genotype data from the 1000 Genomes project as a reference. Briefly, two datasets were combined to select overlapping SNPs, and then pairs of SNPs with a high correlation were pruned with a LD-based algorithm (Greenbaum et al., 2017). Default values of the PLINK 1.9 software (50, 5, 0.2) were set as parameters when calculating LD values (r^2^) between each SNP pair (Greenbaum et al., 2017). The LD was computed for windows which contained 50 SNPs. The SNP with lower frequency of the minor allele was excluded for each pair with *r*^2^ > 0.2. The calculation window was then shifted forward by 5 SNPs. Then repeated above process until each pair of SNPs were in low LD. Finally, 1,783,638 SNPs were included in the following analyses.

### Data Analysis

#### Estimating Heritability and Genetic Correlation

Because the previous FNK-BMD GWAS did not show the proportion of variance explained by the significant findings in their study, the heritability explained by the previous GWAS results was estimated using the GWAS summary statistics. The stratified LD score regression partitioned heritability and estimated the genetic correlation between FNK-BMD and eBMD (Finucane et al., 2015).

#### Conditional FDR Calculation and Conditional Manhattan Plot

Conditional false discovery rate is an extension of the FDR method, which combines the GWAS summary statistics of two phenotypes to evaluate the probability of the association of genetic variants with the principal phenotype conditioned on the strength of the association with the conditional phenotype (Liu et al., 2018). Detailed principles and formulas of the method were described earlier by Greenbaum (Greenbaum et al., 2017). *P*-values from GWAS-derived summary statistics of eBMD and FNK-BMD were searched to identify common SNPs, through SNP pruning for cFDR and conjunction cFDR (ccFDR) analyses. Then we calculated the cFDR for each SNP where FNK-BMD is the principal phenotype conditioned on the strength of association with eBMD (FNK-BMD | eBMD) and vice versa (eBMD | FNK-BMD).

Additionally, ccFDR value was computed to identify genetic variants associated with both eBMD and FNK-BMD simultaneously. The ccFDR value is defined as the probability that the given SNP was wrongly identified to be associated with both phenotypes. The thresholds of cFDR were set at the maximum cFDR with *p* value less than 5 × 10^–8^ in the principal phenotype (Liley and Wallace, 2015). Manhattan plots were created with R software.

#### Pleiotropic Enrichment Estimation

To assess pleiotropy between eBMD and FNK-BMD, conditional QQ and fold-enrichment were plotted. We presented the QQ curves of the quantiles of nominal –log_10_ (*p*)-values obtained from GWAS summary statistics for the association of the SNPs subsets in the conditional phenotype below each significance threshold. The x-axis and y-axis are empirical quantiles of the nominal *p* values (empirical distribution functions) and nominal *p* values (-log_10_ (*p*)), respectively. Herein, nominal -log_10_ (*p*)-values were stratified by *p*-values of conditional phenotype with the cutoffs as *p* < 1 (expected base line, all SNPs), *p* < 0.1, *p* < 0.01, *p* < 0.001 and *p* < 0.0001. A leftward curve defection from the expected base line in the QQ plots represents pleiotropic enrichment shared by two phenotypes.

Nominal *p* values (- log_10_ (*p*)) and fold enrichments were plotted on the x-axis and the y-axis in fold-enrichment plots, respectively. We presented fold-enrichment plots of nominal - log_10_ (*p*)-values for eBMD SNPs below the standard GWAS threshold of *p* < 5 × 10^–8^ and for subsets of SNPs determined by the significance of their association with FNK-BMD and vice versa. A shift above the expected baseline indicates pleiotropy between the two phenotypes (Schork et al., 2013).

### Colocalization Analysis

The Bayesian colocalization method estimates the probability of each genomic locus containing a genetic variant that is causally associated with both traits (Guo et al., 2015). The Bayesian approach summarizes the evidence of each genetic region for five mutually exclusive hypotheses simultaneously:

H_0_: There exists no SNPs in the region that are associated with either trait,

H_1_ (Model 1): There exists one causal SNP in the region that is associated with the first trait,

H_2_ (Model 2): There exists a causal SNP in the region that is associated with the second trait,

H_3_ (Model 3): There exists a single causal SNP in the region that are associated with both traits,

H_4_ (Model 4): There exist two distinct causal SNPs in the region, one for each trait.

Support for each hypothesis is quantified on the basis of posterior probabilities (PP), defined by PP_0_, PP_1_, PP_2_, PP_3_, or PP_4_ (Guo et al., 2015).

Herein, colocalization was analyzed with *gwas-pw* software (Pickrell et al., 2016), which is available at https://github.com/joepickrell/gwas-pw. The method requires the effect size estimates and standard errors of each SNP for the two phenotypes. The whole genome was split into 1,703 non-overlapping regions (blocks). The regional posterior probability of the region containing one SNP causally associated with both phenotypes larger than 0.9 corresponded with a FDR < 0.1. The SNP in each region with the largest posterior probability was defined as putative causal SNP.

### Genes and SNPs Annotation

ANNOVAR software was employed to annotate the SNPs of interest (Alexander et al., 2017). For genes of interest, Gene Ontologies (GO) and Kyoto Encyclopedia of Genes and Genomes (KEEG) pathways were analyzed using DAVID 6.7^1^.

## Results

### Heritability and Genetic Correlation

The previous FNK-BMD GWAS accounted for 9.1% of the SNP-based heritability (95% CI: 8.1–10.1%). The genetic correlation between FNK-BMD and eBMD was estimated to be 0.64 (*p* = 5.9 × 10^–61^).

### Pleiotropy Between eBMD and FNK-BMD

**FIGURE 1:**
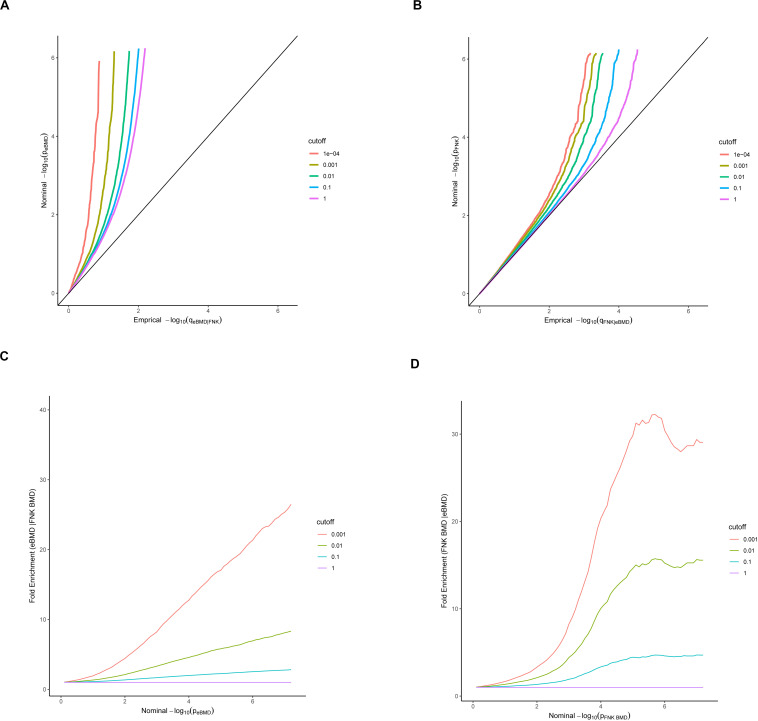
Stratified QQ **(A,B)** and Enrichment **(C,D)** plots. Stratified QQ plots of nominal versus empirical –log_10_ *p*-values in eBMD as a function of significance of the association with FNK-BMD **(A)**, and in FNK-BMD as a function of significance of the association with eBMD **(B)**. Fold-enrichment plots **(C,D)** of enrichment versus nominal –log_10_ *p*-values for eBMD below the standard GWAS threshold of *p* < 5 × 10^–8^ as a function of significance of the association with FNK-BMD, and FNK-BMD below the standard GWAS threshold of *p* < 5 × 10^–8^ as a function of significance of the association with eBMD.

The conditional QQ plot for eBMD conditional on FNK-BMD indicated enrichment of pleiotropic SNPs across the different significance thresholds for FNK-BMD (Figure 1). An obvious upward shift from the expected baseline was detected when restricting the SNPs subgroup with a stronger level of association with the conditional phenotype, indicating an increase in the number of true associations for a given FNK-BMD *p*-value (Figure 1A). Similar enrichment of pleiotropic SNPs for FNK-BMD given eBMD, was evidenced by a similar departure pattern across the different curves (Figure 1B). For any given eBMD nominal *p*-value, a deviation from the null line demonstrated a greater proportion of true pleiotropic associations.

The fold-enrichment plots showed SNP enrichment for eBMD with FNK-BMD across different significant levels, and also for FNK-BMD with eBMD (Figures 1C,D). It illustrated that between the SNPs subgroup with the strongest association in the conditional phenotype and all SNPs groups, the proportion of SNPs associated with the principal phenotype increased by approximately 30-fold at the genome-wide significance level.

### eBMD and FNK-BMD Loci Detected by cFDR

We identified 9,500 SNPs significantly associated with eBMD (cFDR < 9.8E-06), given their associations with FNK-BMD (Supplementary Table S1). All of these SNPs had *p* values less than 1 × 10^–5^ and 8,933 reached the genome-wide significance at *p* < 5 × 10^–8^ in the original GWAS meta-analysis for eBMD (Kemp et al., 2017). Among the 9,500 SNPs we identified, 50.2% (4,769 SNPs) were intronic, 1.7% (*n* = 163) were juxtaposed to the 3’ or 5’ terminals of corresponding genes, and 0.7% (*n* = 71) were in the 3’ or 5’ UTR (Supplementary Table S1).

Conditional on the association of SNPs with eBMD, we identified 137 significant SNPs (cFDR < 8.9E-04) associated with FNK-BMD (Supplementary Table S2). In the original FNK-BMD GWAS meta-analysis (Zheng et al., 2015), 7 SNPs had *p* values less than 1 × 10^–5^ while 4 of them reached the standard genome-wide significance. Among the 137 SNPs identified, 56.2% (77 SNPs) were intronic, and 1.6% were juxtaposed to the 3’ or 5’ terminals of corresponding genes (Supplementary Table S2).

### Pleiotropic SNPs for Both eBMD and FNK-BMD

**FIGURE 2:**
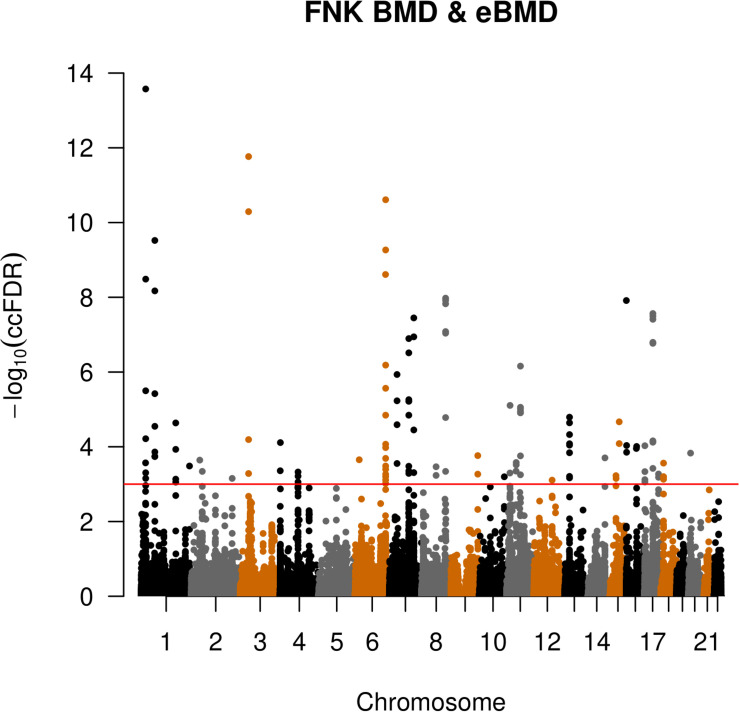
Conjunction Manhattan plot of conjunction –log_10_ FDR values for eBMD and FNK-BMD. The red line marking the conditional –log_10_ ccFDR value of 3.1 corresponds to a conjunction FDR < 8.9E-04.

We identified 124 pleiotropic SNPs that were significantly associated with both eBMD and FNK-BMD using ccFDR method (ccFDR < 8.9E-04) (Supplementary Table S3 and Figure 2). It suggested that most of SNPs (90.5%, $\frac{124}{137}$) associated with FNK-BMD were also associated with eBMD. However, most of SNPs (98.7%, 1-$\frac{124}{9500}$) associated with eBMD were not associated with FNK-BMD. Although, this can be partly explained by the relatively large sample size of eBMD study and the relatively small sample size of FNK-BMD study. Among these SNPs, 25 previously reached the standard GWAS significance (*p* < 5 × 10^–8^) both in the original eBMD and FNK-BMD GWASs. Another 95 pleiotropic SNPs were significantly associated with eBMD in original eBMD GWAS (original *p* < 5 × 10^–8^). Four (rs79676715, rs1381635, rs7816021 and rs4263799) of these statistically significant SNPs identified by ccFDR were not reported by the original eBMD and FNK-BMD GWASs.

The 124 pleiotropic SNPs were located within 73 different genes dispersed over 18 autosomes. GO analyses indicated that these genes were significantly enriched in the canonical Wnt signaling pathway (GO: 0060070, *p* = 3.8E-06) and positive regulation of transcription, DNA-templated (GO: 0045893, *p* = 1.3E-05) (Table 1). KEGG pathway analysis confirmed that pleiotropic genes were significantly enriched in Wnt signaling pathway (hsa04310, *p* = 1.8E-06), and also identified signaling pathways regulating pluripotency of stem cells (hsa04550, *p* = 3.9E-05) and genes associated with basal cell carcinoma (hsa05217, *p* = 1.5E-05) (Table 2).

**TABLE 1:** GO terms enriched by pleiotropic genes.

**Gene Ontology ID: Term**	**Genes**	***p*-value**	**FDR**
**Biological process**			
GO: 0060070 canonical Wnt signaling pathway	WNT4, SMAD3, FZD7, CTNNB1, AXIN1, LRP5	3.78E-06	5.67E-03
GO: 0045893 positive regulation of transcription, DNA-templated	CRTC3, JAG1, SOX6, GLI3, TCF7L2, ATF1, CTNNB1, FUBP3, DDX17, FLI1, TNKS, BCAS3, FGF1, BMP2, WNT4, FUBP3, SOST, ESR1, SMAD3, FZD7, CTNNB1, AXIN1, LRP5, ZNF423	1.29E-05	1.94E-02
			

**TABLE 2:** KEGG pathways enriched by pleiotropic genes.

**Term**	**Genes**	***p*-value**	**FDR**
hsa04310: Wnt signaling pathway	WNT16, WNT4, SOST, FZD7, CTNNB1, AXIN1, LRP5	1.80E-06	1.75E-03
hsa05217: Basal cell carcinoma	WNT16, WNT4, FZD7, CTNNB1, AXIN1	1.50E-05	1.45E-02
hsa04550: Signaling pathways regulating pluripotency of stem cells	WNT16, WNT4, SMAD3, FZD7, CTNNB1, AXIN1	3.94E-05	3.82E-02

### Colocalization Analysis

We identified 37 genomic regions containing a SNP that influences both eBMD and FNK-BMD (model 3) (Supplementary Table S4), 3 genomic regions containing two distinct causal SNPs associated with both traits, respectively (model 4) and 627 regions containing causal SNPs only for eBMD (model 2) (Supplementary Table S5), at a threshold of the posterior probability of model 3 larger than 0.9. The 37 putative causal SNPs annotated to 53 different genes, which were significantly enriched in the biological processes of canonical Wnt signaling (GO: 0060070, *p* = 6.5E-07), positive regulation of transcription (GO: 0045893, *p* = 6.7E-06), and DNA-templated and positive regulation of transcription from RNA polymerase II promoter (GO: 0045944, *p* = 1.6E-05) (Table 3). Block 42 (Genomic region: chr1: 66939404–68477895), containing the candidate gene *GNG12-AS1(WLS)*, was associated with both eBMD and FNK-BMD, and the putative casual SNP (rs2566759) in this region is associated with spine BMD in a previous study (Mullin et al., 2016). Of the 37 genomic regions identified, 26 contained pleiotropic SNPs that were identified in ccFDR analysis. We noted that the putative causal SNPs identified by colocalization analysis were distinct from pleiotropic SNPs in each genomic region, suggesting the ccFDR method is apt to identify the variant associated with both phenotypes instead of the causal one.

**TABLE 3:** GO terms enriched by causal genes.

**Gene Ontology ID: Term**	**Genes**	***p*-value**	**FDR**
**Biological process**			
GO: 0060070 canonical Wnt signaling pathway	SMAD3, FZD7, KLF4, CTNNB1, AXIN1, LRP5	6.49E-07	9.35E-04
GO: 0045893 positive regulation of transcription, DNA-templated	FUBP3, SOST, ESR1, SMAD3, FZD7, KLF4, CTNNB1, AXIN1, LRP5	6.66E-06	9.59E-03
GO: 0045944 positive regulation of transcription from RNA polymerase II promoter	RPS6KA5, FUBP3, TNFSF11, MEOX1, TRPS1, ESR1, SMAD3, SOX6, KLF4, CTNNB1, LRP5	1.60E-05	2.30E-02

## Discussion

In the present study, we combined two GWAS summary statistics by cFDR to identify the pleiotropic and/or novel SNPs associated with eBMD and/or FNK-BMD. We discovered 9,500 SNPs and 137 SNPs for eBMD and FNK-BMD, respectively. It suggested that the majority of SNPs associated with eBMD were not identified for FNK-BMD. Conjunction cFDR analysis identified 124 pleiotropic SNPs that were significantly associated with both eBMD and FNK-BMD. We also identified 37 putative causal SNPs that may underlie both phenotypes using colocalization analysis. It suggested that both BMD phenotype measurements shared some causal genetic variants in some regions, but in most of independent regions (94.5%, $\frac{3+627}{37+3+627}$) the putative causal SNPs for both phenotypes were different.

As osteoporosis is a complex disease with a strong genetic component, scientists have strived to identify SNPs associated with BMD and/or fracture risk. In the present study, we identified 37 candidate SNPs which are causally associated with both eBMD and FNK-BMD. Among these SNPs, SNP rs884205 is located in the 3’UTR of tumor necrosis factor (TNF) receptor super family member 11 a (*TNFRSF11A*), a gene that is a key driver for BMD and fracture risk (Tu et al., 2015). *TNFRSF11A* encodes RANK, a receptor that regulates bone remodeling. Genetic variation within the *TNFRSF11A* gene is associated with BMD in several previous GWASs (Styrkarsdottir et al., 2009). This SNP is associated with osteoporotic fractures in a Chinese population (Guo et al., 2012).

Dozens of studies in pre- and postmenopausal women, as well as in men, reported numerous of genes that are responsible for regulating serum periostin levels and bone microstructure (Pepe et al., 2018). These include genes with polymorphisms in low-density lipoprotein receptor-related protein (LRP5), estrogen-receptor 1 (*ESR1*) and tumor necrosis factor receptor super family member 11 (*TNFRSF11*) (Pepe et al., 2018). Several novel putative causal SNPs were near those genes identified in a previous study, including rs11228240 (*LRP5*), rs2941741 (*ESR1*), and rs78667121 (*TNFRSF11*).

The SNP rs2566752 is an intronic variant located in the wntless Wnt ligand secretion mediator (*WLS*) gene, a known BMD-associated locus that encodes an integral component of the Wnt ligand secretion pathway (Mullin et al., 2016). The Wnt proteins are members of an evolutionarily conserved family of secreted signaling molecules and are critical for prenatal and postnatal bone development (de Groot et al., 2014; Zhong et al., 2015). Variants within the WLS gene are associated with BMD in several previous GWASs (Rivadeneira et al., 2009). A large meta-analysis also identified rs2566752 as the maximally associated variant from the WLS gene region for both lumbar spine and FNK-BMD (Mullin et al., 2016). Interestingly, bone geometric parameters and BMD pleiotropy were previously detected at the WLS locus (Roshandel et al., 2011). These findings suggest that the associations between rs2566752 and BMD are likely mediated through regulatory effects on the *WLS* gene.

Among the 37 putative causal SNPs, five SNPs overlap with enhancer- or promoter-type chromatin in osteoblasts (i.e., rs9594738, rs7587430, rs11023993, rs4776341, and rs71390846) (Kundaje et al., 2015). Interestingly, rs9594738, overlaps a weak enhancer-type chromatin, lies ∼54.7 kb downstream of A-kinase anchor protein 11 (*AKAP11*) and ∼184.7 kb upstream of *TNFSF11*, or commonly known as *RANKL*. The role of *APAK11* in bone biology is unclear, and it has broad expression in many types of cell and tissue (Encode Project Consortium, 2012). However, *RANKL* is well known to be involved in RANKL/RANK/osteoprotegerin signaling, which is one of the key signaling pathways controlling bone resorption and formation (Chen et al., 2018). In a recent study, the CRISPR/Cas9 deletion of rs9594738 and a pleiotropic SNP rs9533090 was shown to reduce *RANKL* expression at both mRNA level and protein level in U2-OS cells (Zhu et al., 2018).

Another putative causal SNP, rs4776341, overlaps with an active enhancer-type chromatin in osteoblasts, is an intronic variant located in the sterile alpha motif domain containing 3 (SMAD3) gene, which is part of the important TGF-β1 signaling pathway for osteoblast and osteoclast differentiation via the TGF-β1 signaling pathway (Yang et al., 2017). Interestingly, the SNP rs4776341 is associated with eBMD and FNK-BMD by the cFDR method. However, in most causal regions, the putative causal SNPs identified by the colocalization analysis were different from the pleiotropic SNPs identified by the cFDR method. This may be because the real causal SNP was removed using an LD-based pruning method, which was employed to remove one SNP of pairs with *R*^2^ value greater than 0.2 during the process of data preparation in the cFDR method. For example, the pleiotropic SNP rs6426749 and putative causal SNP rs34553872 in block 14 (chr1:21736983-23086441) were in strong LD (*r*^2^ = 1.0). The putative causal SNP rs34553872 was removed during SNP pruning.

Investigating a genetic variant influencing multiple phenotypes is informative in several applications. It can uncover the shared genetic mechanisms between closely related phenotypes, as well as the molecular function of a gene (Solovieff et al., 2013). The cFDR approach was applied herein to identify pleiotropic genetic variants. This method is based on the concept that a SNP with a significant effect on one trait is more likely to be associated with another correlated trait, and therefore is more likely to be detected by combining multiple independent studies (Greenbaum et al., 2017). This approach can not only improve the statistical power for detecting novel variants with true associations, but can detect small to intermediate effect sizes (Andreassen et al., 2013). The genetic enrichment observed in conditional QQ plots illustrates that the power of detecting true association effects greatly enhanced as the cFDR value for a given nominal *p* value decreased. Andreassen and colleagues showed that the cFDR method resulted 15–20-fold increase in the number of SNPs under the same FDR threshold of 0.05, compared with the traditional unconditional FDR method (Andreassen et al., 2013). The cFDR method can identify genetic variant associated with the “principal” phenotype, as long as the *p*-value of association with both the “principal” and the “conditional” phenotypes is below a certain *p*-value threshold (Andreassen et al., 2013; Hackinger and Zeggini, 2017). This method can be used as a variant prioritization tool to identify other variants associated with one phenotype by utilizing information of the association with the second phenotype (Hackinger and Zeggini, 2017). In addition, the cross-phenotype effect can be detected by calculating the maximum value of cFDR for two traits.

The colocalization method is based on Z scores of the SNPs from two input studies, testing an excess of shared signals, and identifying a set of variants with evidence for the association with the two traits (Hackinger and Zeggini, 2017). Additionally, we investigated whether causal SNPs are shared by combining information of multiple SNPs in a region by a colocalization analysis (Fortune et al., 2015). The main strength of this method lies in its speed and analytical forms, and the fact that it can use variants’ effect size estimates and standard errors when they are the only data available (Giambartolomei et al., 2014; Pickrell et al., 2016). This approach focuses on the effects of all SNPs, including those that do not reach genome-wide significance (Fortune et al., 2015). Thus, variants of small effect sizes are more readily identified.

The 124 pleiotropic SNPs identified by the ccFDR analysis were located in 37 independent blocks which were defined in colocalization analysis. Among these blocks, 26 overlapped with causal regions identified by colocalization analysis. This analysis implies that the cFDR method is more powerful for detecting pleiotropic SNPs. Although the purpose of colocalization analysis was to identify the putative causal SNPs affecting both phenotypes, most of the pleiotropic regions (belonging to model 3 and model 4) identified by colocalization analysis contained a putative causal SNP affecting both phenotypes, rather than two distinct putative causal SNPs in the region (one for each trait). Both the cFDR and colocalization results suggest that there is a significant genetic correlation between the two phenotypes. Consistent with the results of the genetic correlation analyses, SNPs influencing FNK-BMD were strongly correlated with eBMD (genetic correlation 0.64, *p* = 5.92E-61). The genetic correlation analyses between two traits indicated that the majority of shared genetic determinants remains to be discovered. The causal association between BMD and fracture risk is well established. Herein, we also found that both FNK-BMD and eBMD have a causal effect on fracture.

Estimated BMD is derived based on the equation: 0.002592 × (BUA+SOS)-3.687. Previously eBMD study indicated that eBMD is not an actual measurement of calcaneal BMD (Frost et al., 2000). Although eBMD is highly correlated with DXA-derived BMD, there is still some SNPs which is associated with DXA-derived BMD but not associated with eBMD. Therefore, we concluded that eBMD can be a good proxy of BMD but cannot be a replacement of DXA-derived BMD, especially used for diagnosis.

We suspected the differences in GWAS sample size could have had an impact on the cFDR results. Previous study has performed a large scale GWAS on total body BMD (TB-BMD) and showed that TB-BMD could be a good proxy of FNK-BMD. We performed cFDR method to identify novel variants with pleiotropic effects on eBMD and TB-BMD using TB-BMD GWAS summary statistics which comprised over 66,000 individuals (Medina-Gomez et al., 2018). The results showed that most of SNPs associated with eBMD were not associated with TB-BMD. The cFDR results are mainly affected by the rank of each SNP’s *p* value, not the exact *p* value in the respective GWAS studies of the two traits. Although this can be partly explained by the different sample sizes of the TB-BMD and eBMD datasets, the results should be reasonable and objective when the studied GWASs datasets are used.

In summary, by applying cFDR and colocalization methods, we identified pleiotropic SNPs associated with two risk traits (e.g., eBMD and FNK-BMD) of osteoporotic fracture, and putative causal SNPs that influence both traits. These findings suggested that eBMD cannot be used for a replacement of DXA-derived BMD (e.g., FNK-BMD). These findings may offer novel pathophysiological insight, and uncover potential targets and pathways for the trait-associated loci impacting bone health in osteoporosis.

## Data Availability Statement

The summary statistics analyzed were derived from three publicly available online GWAS datasets: FNK-BMD dataset, eBMD dataset, and fracture dataset.

## Author Contributions

H-WD, PH, and X-HM: study design. PH, X-HM, and XL: data collection and analysis. PH and XZ: drafting manuscript. H-WD, MS, X-HM, QZ, F-YD, and R-LJ: revising manuscript content. PH and X-HM took responsibility for the integrity of the data analysis. All authors contributed to the preparation and approval of the final manuscript.

## Conflict of Interest

The authors declare that the research was conducted in the absence of any commercial or financial relationships that could be construed as a potential conflict of interest.

## Footnotes

1 https://david.ncifcrf.gov/summary.jsp

## Supplementary Material

TABLE S1 eBMD loci identified with cFDR.

TABLE S2 FNK-BMD loci identified with cFDR.

TABLE S3 SNPs identified by ccFDR analysis.

TABLE S4 Colocalization analysis identified 37 significant genomic regions.

TABLE S5 Results for Model 2 and 4.
